# Dermatofibrosarcoma Protuberans: A Case Report

**DOI:** 10.7759/cureus.117687

**Published:** 2026-10-09

**Authors:** Casandra Monserrat Dominguez Sánchez, Irene Ingrid Resendiz Gomez, Liz Soralla Hernandez Ramirez

**Affiliations:** 1 Internal Medicine, Instituto Mexicano del Seguro Social, Mexico City, MEX; 2 Internal Medicine, Mexican Social Security Institute, Mexico City, MEX; 3 Dermatology, Instituto Mexicano del Seguro Social, Mexico City, MEX

**Keywords:** dermatofibrosarcoma protuberans, dermis, rare skin tumor, skin cancers, subcutaneous tissue

## Abstract

Dermatofibrosarcoma protuberans (DFSP) is a rare malignant tumor of the skin and soft tissues that typically arises in the dermis and subcutaneous tissue, sparing the epidermis. It is a fibroblastic neoplasm characterized by the collagen type I alpha 1 gene with the platelet-derived growth factor B-chain gene fusion. Although it can occur at any age, it is most frequently diagnosed between 30 and 50 years of age. Clinically, DFSP presents in a wide variety of forms, ranging from plaques or small nodules to large, irregular masses; it usually manifests as a firm, multinodular, slow-growing lesion, and the term "protuberans" refers to the nodules that form beneath the skin. Because it can easily be mistaken for benign lesions such as dermatofibroma, keloid, lipoma, or even an abscess, diagnosis is often delayed, particularly when the index of suspicion is low. Complete surgical resection remains the cornerstone of treatment, with options including wide local excision and Mohs micrographic surgery with meticulous assessment of deep and peripheral margins. We present the case of a 49-year-old Mexican woman whose lesion was initially excised as a presumed lipoma and histologically interpreted as a histiocytoma with features of a keloid scar; after recurrence, a second biopsy confirmed DFSP. This case highlights the importance of histopathological evaluation of atypical or treatment-resistant scar-like lesions.

## Introduction

Dermatofibrosarcoma protuberans (DFSP) is a rare, slow-growing soft-tissue sarcoma (STS) of the skin, accounting for 1%-1.8% of all STS cases. DFSP predominantly affects young- and middle-aged adults, with a slight male predominance and a higher incidence among Black individuals [1].

DFSP originates in the dermis and is considered a tumor of fibroblastic lineage, characteristically showing diffuse CD34 positivity [2]. More than 90% of cases harbor the t(17;22)(q22;q13) translocation, frequently present as a supernumerary ring chromosome, which fuses the collagen type I alpha 1 gene (COL1A1) with the platelet-derived growth factor B-chain gene (PDGFB) [3]. The resulting COL1A1-PDGFB fusion places PDGFB under the control of the COL1A1 promoter, leading to continuous autocrine activation of the PDGF receptor beta and uncontrolled proliferation of tumor cells [3]. This mechanism is also the basis for targeted therapy with imatinib in unresectable or metastatic disease [4].

It is a locally aggressive tumor with a tendency for local recurrence, but it rarely metastasizes, with metastasis occurring in approximately 1%-4% of cases [1]. DFSP is clinically significant because it can mimic benign lesions such as keloid scars, which commonly delays diagnosis, and because it infiltrates through finger-like projections that extend well beyond the clinically visible margins (subclinical extension), which necessitates wide local excision or Mohs micrographic surgery (MMS) to prevent recurrence.

We present this case to highlight the diagnostic confusion that can arise between recurrent DFSP and a keloid scar, and to emphasize the importance of early histopathological confirmation and complete excision with adequate margins.

## Case presentation

We present the case of a 49-year-old woman, originally from and residing in the State of Mexico, with no significant chronic medical history. Six years before the current presentation, the patient noticed a firm, erythematous nodule with irregular borders on the right anterior chest. She was evaluated by the surgery service, where a lipoma was suspected, and excision with biopsy was recommended. On examination, the nodule was semimobile and measured 4 cm. It was initially asymptomatic, although it occasionally became pruritic and erythematous. The general surgery service performed an excisional biopsy, and the histopathology report described a histiocytoma with features suggestive of a keloid scar.

After a disease-free interval of approximately three years, the scar began to enlarge, with progressive erythema, swelling, and the appearance of at least three nodules. Over the following three years, the lesion continued to grow, reaching 15 cm, and was accompanied by pruritus, erythema, and pain on light contact with clothing.

On current physical examination, the lesion measured 15 × 7 cm and consisted of six confluent nodules with irregular, poorly defined borders, a violaceous erythematous halo, and areas of residual hyperpigmentation. On palpation, the nodules were firm, semimobile, and adherent to the subcutaneous tissue, with no clinical evidence of involvement of deeper structures (Figure 1).

**Figure 1:**
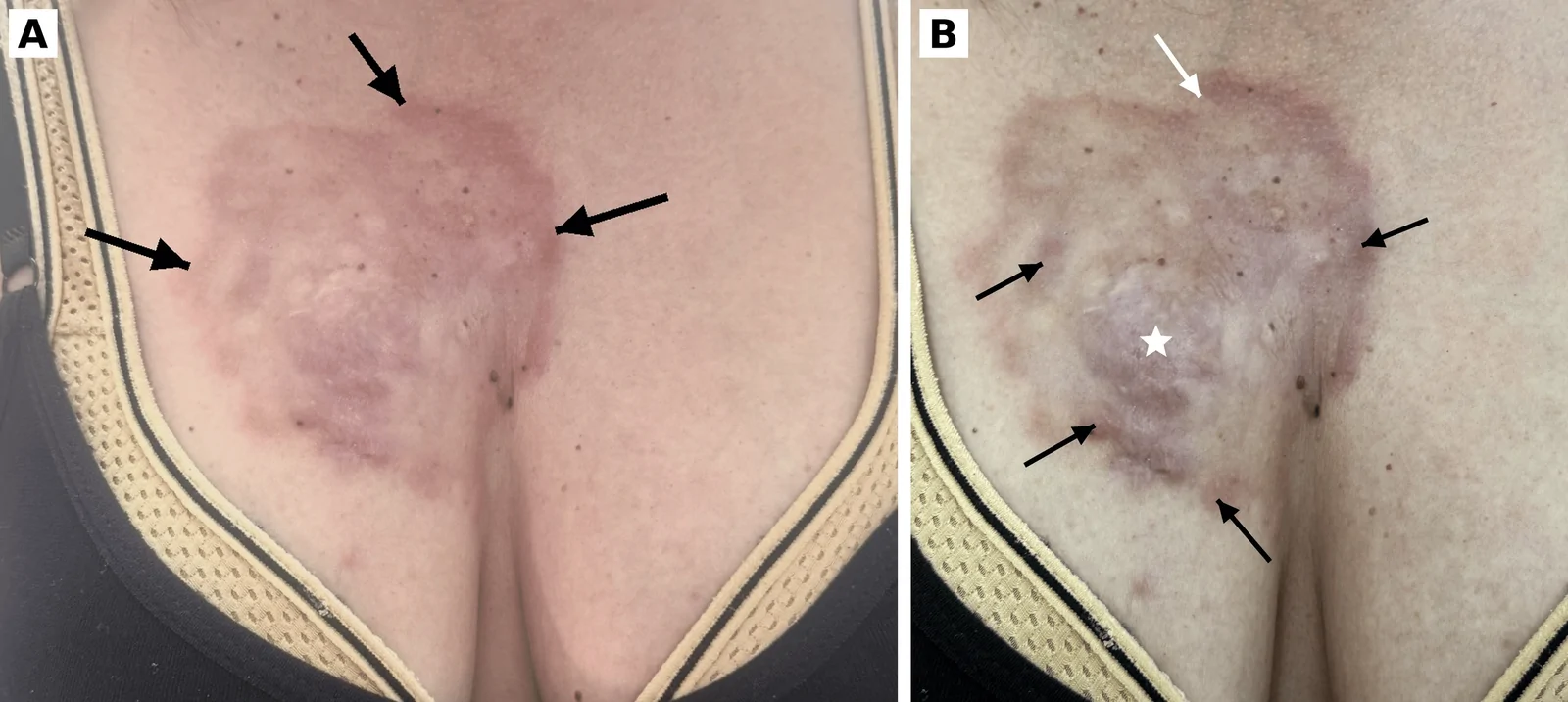
Clinical photographs of the right anterior chest at presentation. (A) Panoramic view showing the location and extent of the lesion, measuring 15 × 7 cm (arrows indicate the lesion borders). (B) Close-up view showing six confluent erythematous-violaceous nodules (black arrows) surrounding a central violaceous scar at the site of the previous excision (white star), with irregular, poorly defined borders and an erythematous-violaceous halo (white arrow)

In March 2026, a second incisional biopsy was obtained from the largest nodule on the right anterior chest. Histopathological examination revealed a densely cellular proliferation of monomorphic spindle cells arranged in a storiform pattern, without marked cytologic atypia (Figure 2), and immunohistochemistry showed diffuse CD34 positivity, confirming the diagnosis of DFSP. Margin status was not assessed, as this was a diagnostic biopsy. The patient was referred to a tertiary care center for staging and definitive surgical treatment, and she is currently under follow-up while awaiting treatment by the surgical oncology service. The clinical timeline is summarized in Table 1.

**Figure 2:**
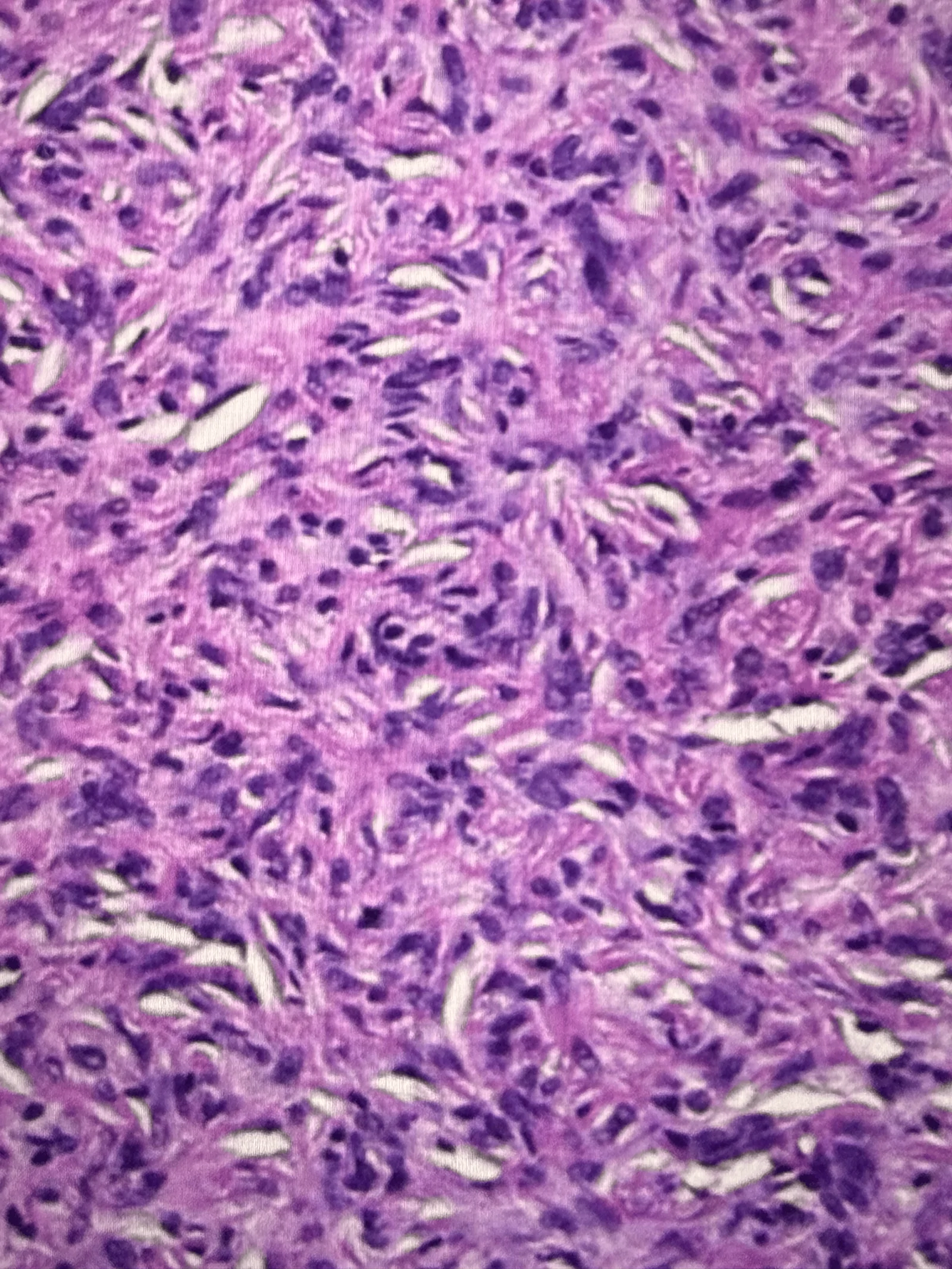
High-power view of the diagnostic biopsy (hematoxylin and eosin) showing a densely cellular proliferation of monomorphic spindle cells arranged in a characteristic storiform pattern, without marked cytologic atypia

**Table 1:** Clinical timeline and diagnostic findings DFSP: dermatofibrosarcoma protuberans

Time point	Event	Findings
Six years before presentation	Firm, erythematous nodule on the right anterior chest; excisional biopsy	Nodule of 4 cm, semimobile; histopathology: histiocytoma with features suggestive of keloid scar
Approximately three years later	Recurrence	Enlargement of the scar with erythema, swelling, and at least three nodules
Following three years	Progression	Growth to 15 × 7 cm; pruritus, erythema, and pain on contact with clothing
March 2026	Second biopsy	Storiform spindle-cell proliferation without marked atypia; diffuse CD34 positivity; diagnosis of DFSP
Current status	Referral to tertiary care center	Awaiting staging and definitive surgical treatment

## Discussion

DFSP serves as a reminder that, in dermatology, appearances can be deceiving. Although the tumor shows relatively indolent biological behavior, with slow growth and a low rate of metastasis, it can easily go unnoticed and, if not adequately treated, has a marked propensity for local recurrence. DFSP affects all races and both sexes, with the highest incidence between 30 and 50 years of age, although congenital and elderly cases have been described [5].

In its early stage, DFSP presents as a small, asymptomatic plaque that may be skin-colored, brownish, pinkish, or purplish, and it is frequently overlooked by the patient or mistaken for a benign lesion. During this slow-growing plaque stage, three clinical variants have been described: the morphea-like type, an indurated plaque that is skin-colored, whitish, or grayish; the atrophoderma-like type, a soft, depressed, atrophic-appearing plaque; and the least frequent angioma-like type, which resembles vascular lesions such as a flat angioma [6]. Over time, the lesion may progress to the nodular or protuberant stage that gives the tumor its name.

The present case illustrates this diagnostic challenge. In our patient, the lesion was initially managed as a presumed lipoma and later interpreted histologically as a histiocytoma with features of a keloid scar, and the correct diagnosis was established only after a second biopsy, six years after the first excision. This scenario is representative of clinical practice, as DFSP can mimic a keloid, dermatofibroma, morphea, lipoma, or even an abscess, and this capacity for mimicry is largely responsible for the diagnostic delay that often accompanies the disease.

Complete surgical excision is the treatment of choice. Because DFSP grows through finger-like projections, it is a highly asymmetric tumor whose subclinical extension can reach a considerable distance from the clinically visible margin, which explains the high recurrence rates after conventional excision with narrow margins [6]. For this reason, wide local excision or MMS is recommended, and the initial surgical intervention is usually the most decisive, as incomplete resection favors recurrence. Targeted therapy with imatinib, based on the COL1A1-PDGFB fusion, is an option for unresectable or metastatic disease [4].

This report has limitations: no imaging studies were performed, molecular confirmation of the COL1A1-PDGFB fusion was not available, and data on definitive surgical treatment and follow-up could not be obtained after the patient was referred to a tertiary care center.

## Conclusions

DFSP is a locally aggressive sarcoma whose ability to mimic benign lesions, particularly keloid scars, frequently delays diagnosis. Our case shows that an initial misdiagnosis and incomplete excision can lead to recurrence and years of diagnostic delay. From a clinical perspective, any keloid-like scar that recurs, enlarges, or develops new nodules after excision, and any persistent, slow-growing plaque that does not clearly fit a benign diagnosis, should prompt a deep biopsy to rule out DFSP. Early histopathological diagnosis allows timely wide local excision or MMS, which remain the cornerstone of treatment and offer the best chance of preventing recurrence.
